# Addressing cancer care in low- to middle-income countries: a call for sustainable innovations and impactful research

**DOI:** 10.1186/s12885-023-11272-9

**Published:** 2023-08-15

**Authors:** D. Cristina Stefan, Shenglan Tang

**Affiliations:** 1 University of Global Health Equity, SingHealth Duke-NUS Global Health Institute, Kigali, Rwanda; 2 SingHealth Duke-NUS Global Health Institute, Duke-NUS, Singapore; 3 Duke Global Health Institute, Duke University, Durham, NC USA; 4 Global Health Research Center, Duke Kunshan University, Kunshan, Jiangsu China

**Keywords:** Cancer control, Cancer research, Low- and middle-income countries

## Abstract

Most new cancer cases are currently arising in low- and middle-income countries, where their outcomes are significantly poorer compared to high-income countries. Innovative solutions are imperiously needed to prevent, detect early, and manage cancer in low- and middle-income countries, aiming to improve the chances of survival.

## Background

Cancer, a global health crisis, knows no boundaries. However, the burden of this devastating disease falls disproportionately on low- and middle-income countries (LMICs). In 2020, LMICs reported estimated age-standardized incidence and mortality rates of all cancers (excluding non-melanoma skin cancer) at 177.6 and 100 per 100,000 population, respectively [1]. Inadequate resources, limited access to specialized care, and fragmented healthcare systems have exacerbated the challenges faced by cancer patients in these regions.

While most high-income countries (HICs) have experienced a decline in mortality rates, largely due to effective cancer screening programs, timely diagnosis, and improved treatments, [2] in most LMICs both cancer incidence and mortality rates have risen. For example, the incidence and mortality of cancer in Africa are expected to double in less than two decades [3].

## Challenges

In addition to the growing burden of known cancers, LMICs are now facing new challenges, such as a rise in early-onset cancer cases among young populations. Breast, cervical, prostate, and colon cancers are among the malignancies that despite showing an early onset, are frequently diagnosed at an advanced stage [4]. Data suggest a higher prevalence of inherited oncogenic mutations in Africa compared to other populations [5, 6]. However, the impact of lifetime exposures to risk factors related to non-inherited forms of cancer remains poorly understood. Meanwhile, cancer survival in LMICs have fallen significantly behind those in HICs. Some of the factors contributing to the lower survival rates are the inadequacy of the screening programs and the poor cancer management.

In LMICs, the concept of implementation research in cancer management is relatively new. The burden of cancer, along with a scarcity of clinicians, fragile infrastructures, and evolving treatment approaches on a global scale, leads to many countries needing significant time to reduce cancer-related mortality. Despite the increasing focus on universal health coverage as part of the UN’s sustainable development goals for 2030, there has been a lack of meaningful research in the financing of cancer care and in the training of health workforce in LMICs. While several leapfrogging initiatives have been proposed for cancer diagnosis and treatment [7], their implementation has been limited so far.

In addition, most of the cancer research is currently centred in HICs. To date, only a minority of cancer patients in LMICs have been involved in clinical trials [8, 9], which are essential in the development and approval of new standards of care to enhance overall survival and quality of life for patients. Furthermore, disparities in the inclusion of women, older adults, and certain racial and ethnic groups persist, hindering the validity of trial findings [10, 11].

Studies exploring the potential connection between climate change and cancer are also primarily conducted in HICs [12, 13]. However, it is crucial to pay equal attention to this issue, considering that global climate disasters disproportionately impact many low-income countries.

Lastly, in recent years, numerous armed conflicts around the world resulted in displaced populations, altered demographics, different environmental exposures and possibly, an increased cancer risk. While researchers have studied the effect of conflict on cancer care [14], little is known about its impact on cancer incidence among the affected population.

## Potential solutions

In order to revolutionize cancer care in LMICs and make a lasting impact on the lives of vulnerable populations, it is crucial to develop new approaches that harness technological advancements, foster aligned partnerships, and implement targeted strategies.

The backbone of effective cancer care relies on robust infrastructures and well-functioning health systems with adequate resources. In LMICs, it is essential to invest in establishing and expanding cancer centres. These centres should be equipped with adequate diagnostic tools, treatment facilities, and a multidisciplinary team of healthcare professionals specialized in oncology.

Additionally, collaboration among governments, philanthropic organizations, and the international community is essential to secure sustainable funding for cancer research, drug access programs, and healthcare providers training. Strengthening infrastructure and strategic resources allocation lay the foundation for improved cancer care in LMICs.

To further enhance progress, innovative partnerships should be established and supported between LMICs and HICs, as well as between countries within the Global South. Such partnerships would foster knowledge transfer, enabling professionals to acquire expertise in specialized cancer care and acquire managerial skills through webinars, virtual meetings and video assisted consultations.

Apart from aiding in healthcare providers training and fostering global connections, new technologies, like telemedicine and mobile health applications, have also the potential to revolutionize cancer care delivery in LMICs. Many patients in remote areas encounter difficulties in accessing specialized care. Telemedicine allows healthcare professionals to offer remote consultations, bridging the gap between patients and oncologists. Mobile health applications can empower patients and caregivers by providing educational resources, symptom monitoring tools, and medication reminders. These technologies can improve patient outcomes, enhance efficiency, and reduce healthcare costs in resource-constrained settings [15].

Tailored approaches are essential to effectively address the challenges of cancer prevention, control, and management in different population groups. Innovative strategies should be designed and implemented, while proven approaches from HICs should be adapted to LMICs. For instance, human papillomavirus (HPV) vaccination for schoolgirls and cervical cancer screening programs, which have shown success in HICs, should be introduced in LMICs. Equitable access to cancer care remains a critical challenge for many LMICs. Identifying existing inequalities, conducting research on their root causes, and developing plans to reduce them are imperative. In this pursuit, the application of Artificial Intelligence (AI) tools, where necessary, can play a significant role in improving access and ensuring fair distribution of resources.

AI holds the potential to overcome the capacity issues faced by LMICs healthcare systems. AI can support, and in some cases even replace, overwhelmed clinical experts by employing automated decision-making based on real-world data inputs. AI might generate effective solutions that cater to the unique characteristics of diverse populations.

As we explore AI’s potential, special attention must be given to cancer workforce training to adapt to the anticipated changes brought about by AI utilization, even though these changes are not yet clearly defined.

Lastly, it is crucial to enhance access to clinical trials to cancer patients from LMICs and ensure equitable participation of women, children, and vulnerable populations. By establishing clinical trials in LMICs, we can facilitate access to cancer medicines that might otherwise be unaffordable, while also fostering global diversification of the clinical research landscape.

In recognition of this important field, we are now welcoming submissions to our new Collection of articles titled ‘Cancer control in low- and middle-income countries’. Of particular interest for our readers would be recent data analysis from country cancer registers, implementation research of prevention and early detection of cancers in LMICs and efficient innovative financing solutions for cancer treatment. More details can be found here: https://www.biomedcentral.com/collections/CCLMIC.

## Data Availability

Not applicable.

## Abbreviations

AI: Artificial Intelligence
HICs: High-income Countries
HPV: Human Papillomavirus
LMICs: Low- and Middle-income Countries
UN: United Nations
